# Influence of Physical Activity on Bone Mineral Content and Density in Overweight and Obese Children with Low Adherence to the Mediterranean Dietary Pattern

**DOI:** 10.3390/nu10081075

**Published:** 2018-08-12

**Authors:** Victoria Muñoz-Hernandez, Lide Arenaza, Luis Gracia-Marco, Maria Medrano, Elisa Merchan Ramirez, Wendy D. Martinez Avila, Maddi Oses, Jonatan R. Ruiz, Francisco B. Ortega, Idoia Labayen

**Affiliations:** 1 PROmoting FITness and Health through physical activity research group (PROFITH), Department of Physical Education and Sports, Faculty of Sport Sciences, University of Granada, 18071 Granada, Spain; mariavmuher@gmail.com (V.M.-H.); lgracia@ugr.es (L.G.-M.); elymerchan@hotmail.com (E.M.R.); wdma@hotmail.com (W.D.M.A.); ruizj@ugr.es (J.R.R.); 2 Nutrition, Exercise and Health Research group, Elikadura, Ariketa Fisikoa eta Osasuna, ELIKOS group, Institute for Innovation & Sustainable Development in Food Chain (IS-FOOD), 18 Public University of Navarra, Campus de Arrosadía, 31006 Pamplona, Spain; laretxo@gmail.com (L.A.); maria.medrano.echeverria@gmail.com (M.M.); maddiosre@gmail.com (M.O.); 3 Growth, Exercise, Nutrition and Development (GENUD) Research Group, Universidad de Zaragoza, 50009 Zaragoza, Spain

**Keywords:** Mediterranean dietary pattern, overweight children, bone health, physical activity

## Abstract

The objective of the present cross-sectional study was to examine the associations of physical activity and the adherence to the Mediterranean dietary pattern (MDP) with bone mineral content (BMC) and density (BMD) in children with overweight and obesity. A total of 177 (*n* = 80 girls) children with overweight and obesity aged 8 to 12 years old participated in the study. Both BMC and BMD were assessed by Dual-Energy X-ray absorptiometry. Dietary patterns were assessed by the KIDMED questionnaire and two 24-hour recalls. Physical activity was assessed by accelerometers for 7 consecutive days (24 hours/day). Low adherence to the MDP was observed in 82.4% of participants. Higher physical activity levels (of at least moderate intensity) and lower sedentary time were significantly associated with BMC and BMD in children with low adherence to the MDP (all *p* < 0.05). No associations were observed between physical activity and BMC and BMD in children with high adherence to the MDP. In conclusion, engaging in moderate to vigorous physical activity and reducing the time spent in sedentary behavior might be particularly beneficial for improving bone health in overweight or obese children with poor adherence to the Mediterranean dietary pattern.

## 1. Introduction

The World Health Organization has recognized osteoporosis as a chronic noncommunicable disease [1]. Osteoporosis is the most common skeletal disease in humans and its prevalence increases with age. Fractures resulting from it are a major public health problem not only in developed countries but also in developing countries [2].

Childhood and adolescence are important life stages characterized by changes in skeletal size and shape [3,4]. In this regard, maximizing peak bone mass is an essential prevention strategy towards osteoporosis, since it is an important predictor of areal bone mineral density (BMD) later in life and approximately 90% of the peak bone mass is attained by the age of eighteen [5]. Bone development across the lifespan has an important genetic component, with epidemiological studies attributing to genes 60–80% of the variability in BMD [6]. However, environmental non-modifiable (e.g., hormones) [7] and modifiable factors (i.e., dietary habits and physical activity (PA)) [8,9,10] are of great importance and account for the remaining variance.

The contribution of PA and exercise on bone development during growth has been extensively documented in the literature [11,12,13,14,15,16]. The benefits of PA on bone health in young people are related to its positive association with lean mass and depends on the ability of the skeleton to adapt to mechanical loading. The latter is explained by the mechanostat theory indicating that “bigger muscles exert higher tensile forces on the bones they attach’’ [17]. In addition, there is evidence that objectively measured sedentary time (ST) is negatively associated with bone mineral density of the lower limbs independently of moderate-to-vigorous PA (MVPA), but not with whole body bone outcomes in children [18].

In regards to diet, the body of knowledge supports an important role of some micronutrients, like calcium and D vitamin as contributors to attaining an optimal peak bone mass in children [15,16,19,20,21,22,23,24]. It should be noted that most of the standard meals consist of a variety of food items with a combination of nutrients that are likely to interact. The effects of individual food items may be too subtle to detect. Therefore, the analysis of the dietary patterns of the population constitutes a more comprehensive approach than the evaluation of dietary exposure in order to examine its relationship to health outcomes.

The Mediterranean dietary pattern (MDP) is characterized by a high consumption of fruits, vegetables, legumes, olive oil, and fish. This dietary pattern is rich in calcium, and includes important micronutrients for bone development. Moreover, it also provides molecules with anti-inflammatory properties and bioactive molecules with antioxidant effects, which contribute to bone development [25,26]. The MPD has shown great benefits in the treatment and prevention of multiple noncommunicable diseases such as obesity and diabetes [27,28,29,30,31,32], but only a few studies have examined its association with bone health in young population [28,29], obtaining nonsignificant findings. By contrast, a positive effect of the adherence to the Mediterranean dietary pattern on BMD has been observed in adult population [30].

To the authors´ knowledge, there is a lack of scientific evidence investigating the effect of PA and the adherence to the MDP on bone outcomes in young population. Therefore, the present study aims to examine the associations of PA and sedentary behavior with bone content and density taking into account the adherence to the MDP in overweight and obese children.

## 2. Methods

### 2.1. Study Design

The current study is based on pooled analyses of baseline data obtained from two randomized control trials: the EFIGRO (ClinicalTrials.gov ID: NCT02258126), and the ActiveBrains (ClinicalTrials.gov ID: NCT02295072) projects. The EFIGRO project was conducted in Vitoria-Gasteiz (North Spain) between September 2014 and June 2017 and the ActiveBrains project was carried out between 2014 and 2017 in Granada (South Spain), respectively. Detailed information about the objectives, design, and protocol has been published elsewhere [33,34]. The study protocols were approved by the Ethic Committee of Clinical Investigation of Euskadi (PI2014045) and the Review Committee for Research Involving Human Subjects at University of Granada (Reference: 848, February 2014). Informed consent was obtained from both participants and their parents or guardians before their enrollment in the study.

### 2.2. Participants

**Figure 1:**
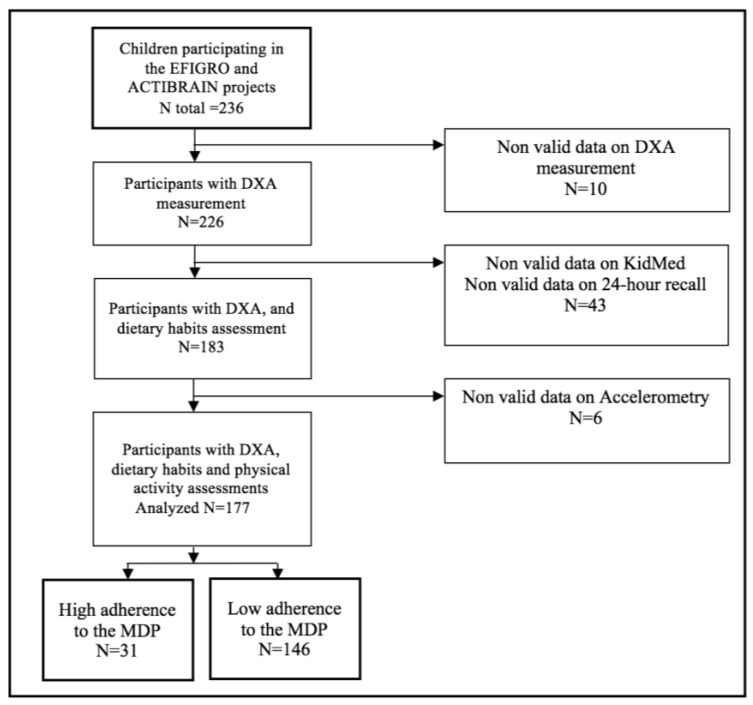
Flowchart of study participants. MDP: Mediterranean dietary pattern. DXA: Dual-energy X-ray absorptiometry.

The current study included a total of 177 children aged 8 to 12 years old (*N* = 80 girls) with overweight or obesity defined according to the World Obesity Federation criteria [35], and with valid data on bone mineral content (BMC), PA, and dietary habits (Figure 1).

### 2.3. Anthropometry and Body Composition

Weight (SECA 760) and height (SECA 220) was measured following standard procedures by the same trained investigator in each study center. Thereafter, BMI and sex- and age-specific BMI z-scores according to the World Health Organization reference standards were calculated. Bone mineral content (BMC, in g), (BMD, in g/cm^2^), body fat percent, and lean mass (LM, in kg) were assessed by Dual-Energy X-Ray Absorptiometry (DXA) using the HOLOGIC Discovery QDR SERIES in ActiveBrains and the HOLOGIC QDR 4500W in EFIGRO.

### 2.4. Dietary Assessment

The KIDMED questionnaire (Mediterranean Diet Quality Index for children and teenagers) was used to evaluate the adherence to the MDP [36,37]. We computed the MDP index using the 10 items directly related to the MDP, while questions about breakfast habit, eating in fast food restaurants, or taking sweets were not included in calculations [38] (Supplemental Table S1). A value of 1 was given to the questions which have a positive connotation in accordance to the MDP. The total MDP score (MDP index) was computed by adding up all the values obtained in the 10 items. Thereby, the MDP index test ranged from 0 to 10 points.

Thereafter, the sample was categorized into two categories according to their MDP index: MDP index ≥8 as high adherence (*N* = 31), and MDP <8 as low adherence (*N* = 146). Moreover, for sensitivity analyses, different cut-off points were used to categorize the adherence to the MDP: (i) equal or above 7 points in the MDP index for high adherence (*N* = 31) and below 7 for low adherence to the MDP (*N* = 146), and (ii) the lowest textile for low adherence and the upper textile for high adherence to the MDP.

Energy intake, calcium, and D vitamin intake were obtained by means of two nonconsecutive 24-hour recalls during a time span of a week by trained dietitians-nutritionists. Thereby, all the foods and drinks consumed on the previous day were recorded. The presence of parents or legal guardians was obligatory for the collection of dietary data due to the difficulty for children to remember recipes or amounts of foods. A book with pictures of different food servings and sizes was used to help the participants to estimate the amount of food consumed. Nutritional composition of the diet was obtained by the Easy Diet software (Xyris software, Brisbane, Australia), supported by the Spanish Association of Dietetics and Nutritionists. The consumption of water and salt was not collected. Neither the children nor the parents knew when they were going to be evaluated so as not to bias the data.

### 2.5. Physical Activity and Sedentary Behavior

ST and PA were objectively assessed with accelerometers worn in to the nondominant wrist (ActiGraph GT3X+, Pensacola, FL, USA) for 7 consecutive days (24 hours/day), except during water-based activities. Children recorded the time when they went to bed, woke up, and removed the device in a diary. We used the mean ENMO (mg) of the day (only waking time) as an indicator of the total PA level. PA intensities (moderate (MPA), vigorous (VPA), and MVPA were estimated using age-specific cut-points for ENMO [39]. All participants with at least 4 days (including 1 weekend day), with ≥10 waking hours, and ≥4 sleeping hours of wearing data were included in the analyses.

### 2.6. Statistical Analysis

Data were analyzed with the use of SPSS program statistical package for Windows software version 24.0 (IBM Corp, Armonk, NY, USA). Data were checked for normality with the use of distribution plots and the Kolmogorov–Smirnov test and all the variables had a normal distribution. For all the analyses we considered *p* < 0.05 as statistically significant. There were no sex interactions on the influence of PA and MDP on bone outcomes, therefore, all the analysis were conducted in boys and girls together. In contrast, it was observed a significant interaction effect between the adherence to the MDP and PA on BMD, when the PA × MDP index interaction term was included in the regression analyses (*p* = 0.015). Then, the sample was divided into high (MDP index ≥8) and low adherence (MDP index <8) to the MDP. Differences in MPA, MVPA, VPA, and total PA between children with low and high adherence to the MDP were examined by *t*-Student tests. Then, the associations of the different levels of PA (MPA, MVPA, VPA, and Total PA) and ST with BMC and BMD were examined by regression analyses adjusting with a priori covariates such as age, sex, study center, height, lean mass, and total energy intake. As sensitivity analyses, all the analyses were repeated using other cut-points to categorize children as having low adherence to the MDP (MDP <7: High MDP *N* = 51 and Low MDP *N* = 126) and lower vs. upper tertile of MDP. Moreover, the analyses were repeated including pubertal stage instead of age into the regression models and further adjusting for calcium and D vitamin intake.

## 3. Results

Table 1 shows the main characteristics of the study sample according to their adherence to the MDP. Having high adherence to the MDP was more frequent in girls than in boys (*p* < 0.05). However, there were no significant differences in the MDP index between girls and boys (Supplemental Table S1). Age, puberty stage, BMI, BMI *z*-score, body fat percent, lean mass, and bone mineral content and density in whole body, as well as in the upper and lower limbs did not significantly differ between children having high or low adherence to the MDP (Table 1).

**Figure 2:**
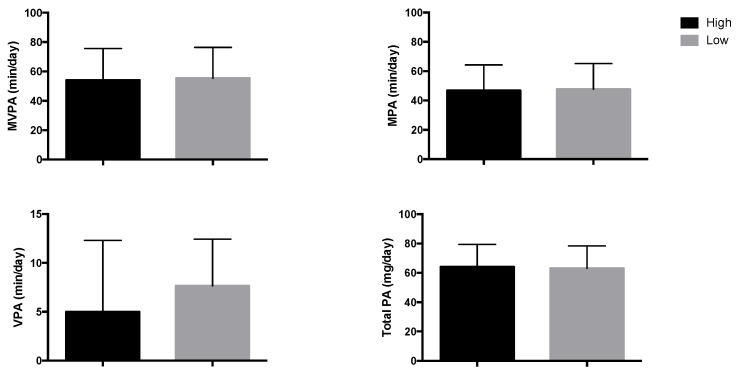
Moderate physical activity (min/day), moderate–vigorous PA (min/day), vigorous PA (min/day), and total PA (mg/day) for the sample divided in high or low adherence to the Mediterranean dietary pattern. Analyses were examined by *t*-Student tests and no significant differences were found. High adherence to the Mediterranean diet pattern index: MDP index ≥8; Low adherence to the Mediterranean dietary pattern: MDP index <8.

There were no significant differences in the adherence to the MDP (*p* = 0.188) or in the number of participants having high adherence to the MDP between the two study centers (*p* = 0.169). Demographic, bone health, PA, and dietary intake characteristics of participants according to the study center can be found in Supplemental Table S2. There were no significant differences in time spent in MPA (*p* = 0.796), MVPA (*p* = 0.773), PA (*p* = 0.761), and VPA (0.737) in children with high or low adherence to the MDP (Figure 2).

PA levels were significantly associated with higher BMC (B = 1.169, age, sex, study center, height, and lean mass adjusted, *p* = 0.001) and BMD (*r* = 0.184, adjusted *p* = 0.001), while there were no significant associations of the adherence to the MDP with BMC and BMD (both adjusted, *p* > 0.1). However, in children with high adherence to the MDP, there were no significant associations of PA, MPA, MVPA, VPA, and ST with BMD and BMC-related outcomes (Table 2). In contrast, higher levels of PA, MPA, MVPA, and VPA were associated with higher BMC and BMD in the whole body (all *p* < 0.01), while ST was negatively associated with both BMC and BMD (*p* < 0.05) in children with low adherence to the MDP. The associations of PA variables and ST with BMC and BMD in the upper and lower limbs showed similar trends (Table 2).

All the analyses were repeated using other cut-points to categorize children as having low adherence to the MDP (MDP <7: High MDP *N* = 51 and Low MDP *N* = 126) and lower vs. upper textile of MDP and the results did not change. Also, the analyses were repeated where pubertal status was used instead of age into the regression models and after further adjustment for calcium and D vitamin intake and the results did not substantially change.

## 4. Discussion

We examined the influence of PA and the adherence to the MDP on BMC and BMD in overweight and obese children. The results show that PA is associated with both BMC and BMD in those children who had low adherence to the MDP, whereas no associations were observed among children with high adherence to this dietary pattern. These findings confirm previous studies and further reinforce the role of PA on bone health, especially in those children with a poor dietary pattern. To our knowledge, this is the first study examining the interaction between PA and adherence to MDP on children’s bone health in children, which is hampered across study comparisons.

Regular PA in prepubertal growth period is one of the most important factors influencing peak bone mass, which is line with our results showing that higher PA levels were associated with increased BMC and BMD. Longitudinal and interventional studies with PA reported mixed results. A meta-analysis by Specket et al. [40] including 22 cross-sectional trials concluded that PA increased the bone mass of infants aged 3 to 18 years. Similarly, Davies et al. [22] examined the influence of genetic, fetal, and environmental factors on the acquisition of bone mass in children, and reported that PA was the most beneficial factor to increase bone mass during growth. In a two year follow-up longitudinal study, it was observed that MVPA and VPA had significant positive effects on femoral neck BMD, but not in whole body BMD. In adolescents, Gabel et al. reported that VPA frequency was positively associated with bone strength measured with high-resolution peripheral quantitative computed tomography scans (HR-pQCT) across adolescence [41]. Similarly, in other longitudinal studies from childhood to early adulthood, MVPA was a positive independent predictor of bone strength assessed by HR-pQCT [42]. In a systematic review and meta-analysis by Chaplais et al. [43] examining the influence of intervention studies with exercise on bone health in adolescents with obesity, it was reported that although beneficial effects on general health were observed, structured intervention of PA did not seem to influence bone metabolism markers.

In childhood, the time spent in MVPA decreases, while the amount of time on sedentary behaviors is increasing, which has independent adverse effects on cardiovascular health [44]. The few studies examining the association of objectively ST with bone outcomes in children reported either no significant effects on whole body BMC and BMD [45,46] or small positive associations of ST with BMC [47]. However, none of these studies were focused on overweight children, and the influence of dietary habits and ST on bone health was not studied, which hamper comparisons. In the current study, lower ST was associated with higher whole body BMC and BMD in those participants with low adherence to the MDP. These results suggest that reducing the time in sedentary behaviors in children with overweight/obesity with poor dietary habits may have positive effects on their bone accrual. However, these results should be taken with caution due to the small size.

We observed no association between MDP and bone-related markers in our population of overweight and obese children, which concur with previous studies. Julian et al. [29] and Monjardino et al. [48] observed no association of the MDP assessed by means of two 24-hour recall and the KIDMED with and BMC and aBMD in adolescents. Of note is that in the Monjardino et al. [48] study, they excluded those items from the KIDMED that had no relation with the Mediterranean diet, as we did it in the current study. The Mediterranean diet is characterized by a high intake of fruits, vegetables, whole grains, legumes, nuts, fish, and virgin olive oil [25]. This pattern favors alkalysis, appropriate to stimulate bone mass metabolism [49]. Furthermore, magnesium promotes good metabolism of calcium [50], and potassium increases calcium retention by the kidneys [51]. Fish is rich in PUFAs, especially *n*-3, which has anti-inflammatory properties, of vital importance for bones. Likewise, Prynne et al. [26] observed the positive effect on the adolescent bone mass of the consumption of fruits and vegetables. A longitudinal study [52] in children aged 4 to 8 years showed that a diet rich in dark-green and deep-yellow vegetables was directly associated with high bone mass. On the other hand, it also contains ingredients that increase the acidity of the medium such as cereals, legumes, and nuts that would have the opposite effect on bone [53]. Seiquer et al. [54] reported that a varied diet based on the MDP improved dietary calcium utilization, and consequently, might help to maximize the peak bone mass in male adolescents.

It has been reported that higher time in sedentary behaviors may result in greater bone resorption leading to reduced BMC, whereas PA and healthy dietary habits are important environmental factors that increase bone mineral accrual during growth. Furthermore, our results suggest that an increase of 1 hour/day of the time in MVPA and a reduction of 48 min/day of the time spent in sedentary behavior in overweight children with low adherence to the MDP may result in an increase of 10 g in BMC. As one of the major preventive strategies of bone health is the optimization of peak bone mass in the growing period, our results on the influence of PA and ST in children with low adherence to the MDP suggest that intervention programs targeting on increasing PA levels and reducing the time on sedentary behaviors may be of particular interest in children with overweight or obesity whose lifestyle patterns are characterized by their poor dietary habits, low PA levels, and high ST [55].

The current study has several limitations. The most important limitation is the relatively small sample size and, particularly, the low number of children classified in the group with high adherence to MDP; this could be due to the fact that our sample is exclusively composed of overweight and obese children whose dietary habits are unhealthier than in nonoverweight youth. Moreover, the cross-sectional design of the study does not allow us to establish causality, and therefore these findings should be taken with caution.

## 5. Conclusions

In conclusion, the current study shows that higher PA levels and a lower time in sedentary behaviors are associated with bone health in overweight or obese children with poor adherence to the Mediterranean dietary pattern. These results suggest that engaging in moderate to vigorous PA and reducing the time spent in sedentary behaviors might be particularly beneficial for improving bone health in overweight or obese children with poor adherence to the Mediterranean dietary pattern. Future exercise-intervention studies are needed to confirm whether exercise is able to have a positive impact on children’ bone health independently of their adherence to a Mediterranean dietary pattern.

## Figures and Tables

**Table 1:** Characteristics of participants according to their adherence to the Mediterranean dietary pattern (MDP).

	High Adherence to the MDP (*N* = 31)		Low Adherence to the MDP (*N* = 146)		*p* ^1^
	Mean	SD	Mean	SD	
Girls (*n*, %)	19, 61.3		61, 41.8		0.047
Age (years)	10.2	1.2	10.4	1.2	0.495
Puberty stage (*n*) ^2^ I and II III, IV and V	21, 10		105, 37		0.697
Height (cm)	146.4	8.8	145.2	8.0	0.444
Weight(Kg)	56.6	8.7	55.0	10.8	0.385
Body mass index (Kg/m^2^)	26.4	3.2	25.9	3.4	0.458
Body mass index *z*-score	2.8	0.9	2.7	0.8	0.591
Body fat percent	41.6	5.8	41.1	4.7	0.657
Lean mass (Kg)	31.0	4.7	30.5	5.1	0.598
Areal Bone Mineral Density (g/cm^2^)					
TBLH	0.80	0.05	0.80	0.06	0.801
Upper limbs	0.62	0.05	0.62	0.05	0.859
Lower limbs	0.95	0.07	0.95	0.09	0.993
Bone Mineral Content (g)					
TBLH	1025.4	179.8	999.2	203.3	0.476
Upper limbs	87.0	17.5	85.2	18.3	0.607
Lower limbs	259.0	50.2	255.7	56.2	0.745
Dietary characteristics					
Energy intake (kcal/day)	1806	384	1769	378	0.630
MDP index	8.6	0.7	5.1	1.3	<0.001
Calcium intake (mg/day)	660	217	661	226	0.968
D vitamin intake (µg/day)	1.92	2.23	1.87	1.97	0.902

^1^ Analyzed by Student *t*-test, ^2^ Some missing data in the low adherence groups *N* = 4. TBLH: total bone mineral content of body less head. Upper limbs: average of both arms. Lower limbs: average of both legs. MDP index: Adherence to the Mediterranean dietary pattern index, High adherence to the Mediterranean diet pattern index: MDP index ≥8; Low adherence to the Mediterranean dietary pattern: MDP index <8; SD: standard deviation.

**Table 2:** Linear regression models addressing the associations of physical activity with bone mineral density and content according to the adherence to the Mediterranean dietary pattern in children with overweight/obesity.

	Total Body Less Head			Upper Limbs			Lower Limbs		
	B	95% CI	*p*	B	95% CI	*p*	B	95% CI	*p*
Areal Bone Mineral Density (g/cm^2^)									
High adherence to the Mediterranean dietary pattern (*N* = 31)									
MPA (min/day)	0.115	−0.001; 0.002	0.52	0.087	−0.001; 0.001	0.651	−0.001	−0.001; 0.001	0.997
MVPA (min/day)	0.084	−0.001; 0.001	0.630	0.051	−0.001; 0.001	0.784	−0.012	−0.001; 0.001	0.93
VPA (min/day)	−0.018	−0.004; 0.003	0.911	−0.057	−0.004; 0.003	0.739	−0.040	−0.005; 0.003	0.768
ST (min/day)	−0.015	0.000; 0.000	0.942	0.072	0.000; 0.000	0.735	−0.001	0.000; 0.000	0.996
Total PA (mg/5 sec)	0.030	−0.001; 0.001	0.871	−0.049	−0.001; 0.001	0.802	−0.016	−0.002; 0.001	0.918
Low adherence to the Mediterranean dietary pattern (*N* = 146)									
MPA (min/day)	0.169	0.004; 0.001	0.004	0.203	0.000; 0.001	0.007	0.123	0.000; 0.001	0.027
MVPA (min/day)	0.185	0.000; 0.001	0.002	0.204	0.000; 0.001	0.007	0.142	0.000; 0.001	0.011
VPA (min/day)	0.183	0.001; 0.004	0.002	0.142	0.00; 0.003	0.062	0.167	0.001; 0.005	0.002
ST (min/day)	−0.150	0.000; 0.000	0.012	−0.140	0.000; 0.000	0.072	−0.109	0.000; 0.000	0.056
Total PA (mg/5 sec)	0.210	0.000; 0.001	<0.001	0.206	0.000; 0.001	0.006	0.172	0.000; 0.002	0.002
Bone Mineral Content (g)									
High adherence to the Mediterranean dietary pattern (*N* = 31)									
MPA(min/day)	0.175	−0.512; 4.112	0.121	0.063	−0.215; 0.341	0.642	0.205	−0.127; 1.306	0.102
MVPA (min/day)	0.146	−0.642; 3.088	0.188	0.026	−0.200; 0.243	0.844	0.176	−0.158; 0.995	0.146
VPA (min/day)	0.037	−6.265; 8.928	0.720	−0.82	−1.150; 0.576	0.498	0.071	−1.637; 3.070	0.535
ST (min/day)	−0.062	−0.805; 0.498	0.630	0.006	−0.074; 0.076	0.969	−0.137	−0.295: 0.105	0.335
Total PA (mg/5 sec)	0.118	−1.379; 4.118	0.313	0.018	−0.301; 0.342	0.896	0.162	−0.317;1.375	0.209
Low adherence to the Mediterranean dietary pattern (*N* = 146)									
MPA(min/day)	0.100	0.322; 1.998	0.007	0.159	0.063; 0.269	0.002	0.102	0.083; 0.573	0.009
MVPA (min/day)	0.109	0.355; 1.754	0.003	0.159	0.050; 0.223	0.002	0.112	0.096; 0.504	0.004
VPA (min/day)	0.108	1.509; 7.637	0.004	0.098	−0.013; 0.760	0.058	0.113	0.431; 2.220	0.004
ST (min/day)	−0.092	−0.509; −0.056	0.015	−0.205	−0.083; −0.029	<0.001	−0.064	−0.121; 0.013	0.114
Total PA (mg/5 sec)	0.126	0.723; 2.596	0.001	0.182	0.101; 0.333	<0.001	0.116	0.148; 0.700	0.003

MPA: moderate physical activity, MVPA: moderate–vigorous physical activity, VPA: vigorous physical activity, ST: sedentary time, total PA: total physical activity. B refers to the standardized regression coefficient; 95%CI refers to those from the unstandardized regression coefficient. Adjusted for age, sex, study center, height, lean mass, and total energy intake and energy intake.

## Supplementary Materials

The following are available online at http://www.mdpi.com/2072-6643/10/8/1075/s1, Table S1: Adherence to the Mediterranean dietary pattern (MDP) in boys and girls; Table S2: Descriptive characteristics of the sample by study centre.

## Abbreviations

The following abbreviations are used in this manuscript:

BMD	Body mass density
BMC	Body mass content
LM	Lean mass
MDP	Mediterranean dietary pattern
DXA	Dual-Energy X-ray Absorptiometry
PA	Total physical activity
MPA	Moderate physical activity
MVPA	Moderate to vigorous physical activity
VP	Vigorous physical activity
ST	Sedentary time
