# Supplementary material for: Influence of Physical Activity on Bone Mineral Content and Density in Overweight and Obese Children with Low Adherence to the Mediterranean Dietary Pattern
Source: Nutrients. 2018 Aug 12;10(8):1075. doi: 10.3390/nu10081075 (PMC6116035; doi:10.3390/nu10081075)
Supplement: Supplementary file 1 [file nutrients-10-01075-s001.pdf]

Supplemental Table 1: Adherence to the Mediterranean dietary pattern (MDP) in boys and girls.

	**Boys (N=97)**	**Girls (N=80)**	**P***
Takes a fruit or fruit juice every day (N, %)[a,b, c]	69;71.1	54;67.5	0.604
Has a second fruit every day (N, %)[a,b,c]	41;42.3	36;45	0.717
Has fresh or cooked vegetables regularly once a day (N, %)[a,b,c]	60;61.9	53;66.3	0.547
Has fresh or cooked vegetables regularly more than once a day (N, %)[a,b,c]	28;28.9	25;31.3	0.732
Consumes fish regularly (at least 2-3/week) (N, %) [a,b,c]	64;66	58;72.5	0.354
Likes pulses and eats them >1 week (N, %)[a,b,c]	68;70.1	64;80	0.129
Consumes pasta or rice almost every day (5 or more per week) (N, %)[a,b,c]	28;28.9	26;32.5	0.604
Has cereals/grains (bread, etc.) for breakfast (N, %)[a,b,c]	70;72.2	57;71.3	0.894
Consumes nuts regularly (at least 2-3/week) (N, %)[a,b,c]	25;25.8	21;26.3	0.943
Uses olive oil at home (N, %)[a, b,c]	95;97.9	77;96.3	0.503
Goes ≥1/week to a fast food restaurant (hamburger) (N, %) [a,d]	18,	11,	0.422
Skips breakfast (N, %)[a,d]	9, 9.3	16, 20	0.051
Has a dairy product for breakfast (N, %)[a,c]	90,	75,	1.000
Has commercially baked goods/pastries for breakfast (N, %)[a,d]	49	48	0.227
Takes two yoghurts and/or some cheese (40g) daily (N, %)[a,c]	51	29	0.034
Takes sweets and candy every day (N, %)[a,d]	9	5	0.580
KIDMED score (mean, SD)	6.2, 2.3	6.2, 2.1	0.902
MDP index (mean, SD)	5.6, 1.7	5.9, 1.9	0.389

[a] Included in the calculation of the KIDMED score. [b]Included in the calculation of the MDP index. SD: standard deviation. [c]Scoring positive (+1). [d]Scoring negative (-1). *Analyzed by Chi-squared test. MDP index: Mediterranean dietary pattern score.

Supplemental Table 2. Descriptive characteristics of the sample by study centre.

	ActiveBrains			EFIGRO		
	N	Mean	SD	N	Mean	SD
	82			95		
Girls (n, %)	28 (34.1%)			52 (54.7%)		
Age (years)	82	9.9	1.2	95	10.7	1.1
Height (cm)	82	144.3	7.9	95	146.3	8.2
Weight(Kg)	82	56.2	10.5	95	54.5	10.4
Body mass index (Kg/m^2)	82	26.7	3.4	95	25.3	3.1
Lean mass (Kg)	82	29.7	4.8	95	31.4	5.1
**Areal Bone Mineral Density (g/cm^2)**						
TBLH	82	0.77	0.055	95	0.81	0.060
Upper limbs	82	0.60	0.037	95	0.64	0.051
Lower limbs	82	0.91	0.074	95	0.99	0.078
Bone Mineral Content (g)						
TBLH	82	974.6	192.7	95	1029.0	202.3
Upper limbs	82	83.1	16.3	95	87.6	19.4
Lower limbs	82	248.4	55.5	95	263.1	54.1
**Physical activity (min/day)**						
Moderate	82	46.6	16.9	95	48.3	17.9
Moderate to vigorous	82	54.5	20.4	95	55.7	21.7
Vigorous	82	7.9	4.4	95	7.3	5.1
Sedentary	82	521.8	56.1	95	540.7	75.3
Total (mg/5sec)	82	63.7	14.9	95	62.9	15.8
Energy intake (kcal/day)	82	1698	351	95	1831	395
MDP index	82	5.95	1.89	95	5.58	1.72
High adherence (n, %)*	18(22%)			13(13.7%)		
Low adherence (n, %)	64(78%)			82(86.3%)		

TBLH, total bone mineral content of body less head, Upper limbs: average of both arms Lower limbs: average of both legs; MDP index: Adherence to the Mediterranean dietary pattern index, * High adherence to the Mediterranean diet pattern index: MDP index ≥ 8; Low adherence to the Mediterranean dietary pattern: MDP index< 8; SD: standard deviation.
